# Food Retail Environments in Greater Melbourne 2008–2016: Longitudinal Analysis of Intra-City Variation in Density and Healthiness of Food Outlets

**DOI:** 10.3390/ijerph17041321

**Published:** 2020-02-19

**Authors:** Cindy Needham, Liliana Orellana, Steven Allender, Gary Sacks, Miranda R. Blake, Claudia Strugnell

**Affiliations:** 1 Institute for Health Transformation, Global Obesity Centre, Deakin University, Geelong 3220, Australia; steven.allender@deakin.edu.au (S.A.); gary.sacks@deakin.edu.au (G.S.); miranda.blake@deakin.edu.au (M.R.B.); claudia.strugnell@deakin.edu.au (C.S.); 2 Faculty of Health, Biostatistics Unit, Deakin University, Geelong 3220, Australia; l.orellana@deakin.edu.au

**Keywords:** food retail, food environment, diet, obesity, urban growth

## Abstract

Obesity prevalence is inequitably distributed across geographic areas. Food environments may contribute to health disparities, yet little is known about how food environments are evolving over time and how this may influence dietary intake and weight. This study aimed to analyse intra-city variation in density and healthiness of food outlets between 2008 and 2016 in Melbourne, Australia. Food outlet data were classified by location, type and healthiness. Local government areas (LGAs) were classified into four groups representing distance from the central business district. Residential population estimates for each LGA were used to calculate the density of food outlets per 10,000 residents. Linear mixed models were fitted to estimate the mean density and ratio of ‘healthy’ to ‘unhealthy’ food outlets and food outlet ‘types’ by LGA group over time. The number of food outlets increased at a faster rate than the residential population, driven by an increasing density of both ‘unhealthy’ and ‘healthy’ outlets. Across all years, ratios of ‘unhealthy’ to ‘healthy’ outlets were highest in LGAs located in designated Growth Areas. Melbourne’s metropolitan food environment is saturated by ‘unhealthy’ and ‘less healthy’ food outlets, relative to ‘healthy’ ones. Melbourne’s urban growth areas had the least healthy food environments.

## 1. Introduction

Worldwide obesity prevalence has tripled since 1975 and no country has managed to reverse this trend [1]. Unchecked, projections estimate that more than half of the world’s adult population will be overweight (estimated 2.16 billion) or obese (estimated 1.12 billion) by 2030 [2].

Increasing prevalence of obesity has been linked to supply-side drivers within the global food system, with increased production, retail, marketing and subsequent consumption of high energy, nutrient poor and highly palatable convenience foods [3,4]. Over time, this has contributed to rapid dietary changes. In addition, the different ways in which the global food system interacts with local environmental factors have contributed to the wide variation in obesity prevalence between populations [4]. Indeed, food environments, defined as the “collective economic, policy and social surroundings, opportunities and conditions that influence people’s food and beverage choices and nutritional status” [5] have been identified as a key influence on dietary behaviors and obesity prevalence [6,7]. Global recommendations indicate that governments should play a leading role in addressing these supply-side drivers by introducing fiscal measures and policies around promoting food products consistent with a healthy diet, in an effort to create health-promoting environments [3]. The International Network for Food and Obesity/non-communicable diseases Research, Monitoring and Action Support (INFORMAS) suggest that governments across the globe should implement policies and programmes “to support the availability of healthy foods and limit the availability of unhealthy foods in communities and in-store (product density)” [5]. Local governments in England have been among the first to initiate recommendations, developing proposals to regulate the proliferation of takeaway food outlets through urban planning [8].

Although empirical evidence of a causal relationship between food retail environments (food environments) and obesity is currently limited [9], several US studies have demonstrated associations between the food environment, diet and obesity among adults (≥18 years of age) [10]. In Australia, high proportions of unhealthy relative to healthy food retail outlets (food outlets) have been associated with higher Body Mass Index (BMI) in children [11], adults [12,13] and less healthy dietary factors in adults [14]. Evidence also suggests that fast-food outlet exposure is associated with obesity in Australia [15,16,17]. Variations in food environments across geographic areas [18,19,20,21] have been reported that could help explain disparities in adult obesity prevalence.

Contrasting findings from studies examining the food environment emphasise the need to examine the food environment holistically [21]. The most common food outlet type to be measured is supermarkets and fast-food, leaving a vast array of food outlet types unaccounted for [21]. Whilst absolute measures of access to food outlets (i.e., only a single type, or comparison of two types of food outlets), such as supermarkets, may provide some understanding of the availability of fruit and vegetables within an area, access to other food sources in the broader food environment is needed to identify disparities.

More holistic measures of the food environment, such as healthiness scores, relative measures or dichotomous (healthy or unhealthy) classifications, have been used in recent studies [3,4]. Although few studies have utilized relative measures, those that have report consistent findings in the expected direction (i.e., healthier food environments associated with healthier dietary factors) more often than those using absolute measures. This suggests that the ratio of healthy to unhealthy food stores could be a better predictor of food purchasing and consumption behaviours, and that getting this balance right may be key to prevention efforts [14,21,22]. However, using a dichotomous classification requires classifying food outlets into either category, which often leads to exclusion of food outlets that do not meet either criteria, reducing the validity of studies seeking to determine the influential nature of the food environment [5,6,7]. In our study, the Food Environment Score represents a holistic food environment classification tool, where all food outlets are given a score ranging from −10 to +10 [22]. This novel approach overcomes the limitations of dichotomous classification and enables researchers to examine the food environment as a whole [23].

With rapid growth in urban populations being observed globally, increasing numbers of people are living around cities [24]. Evidence suggests that communities living in new development areas absorbing population growth around cities may face limited access to resources, with establishment of shops and services often withheld until sufficient population densities are present [25,26,27]. With regard to the food environment, little is known about how it is evolving over time alongside population density, particularly in Greenfield sites supporting urban growth. The United Nations emphasises the need for sustainable management of urban growth to ensure the benefits of rapid urbanisation are universally enjoyed, and disparities do not emerge [24]. Numerous studies have sought to identify whether disparities in spatial access to food within the food environment exist across various measures at a single time point [28,29]. Despite the growing number of studies, the evidence as to the mechanism behind disparities forming within food environments is inadequate, emphasising the need for longitudinal studies which are currently lacking [21]. 

Australia’s major cities are experiencing rapid population growth and expansion. For example, the state of Victoria, in Australia’s south-east, has experienced growth of almost 30% between 2008 and 2018 [30] with the population projected to almost double from 4.5 million in 2017 to 8 million by 2050 [31,32]. Of Victoria’s 79 local government areas (LGAs), the Victorian Department of Environment, Land, Water and Planning expect half of this growth to occur in six LGAs on Melbourne’s urban fringe designated as ‘Victorian Growth Areas’ (Growth Areas) [33]. State government strategic documents set planning standards for Growth Areas including that at least 80% of households should be within 1 km of a town center (an important community focal point with a mix of uses to meet local needs) where there is provision for a supermarket; and, within reach by public transport of a “viable convenience store” [34]. The state government is currently yet to provide similar standards for ‘established’ LGAs (n = 73) regarding access to supermarkets or other food outlets.

Growth Areas experience higher rates of obesity than established areas of Greater Melbourne (Melbourne), with food environment disparities increasingly being examined to determine their potential contribution to the disproportionate prevalence of people with obesity [26,35]. In 2013, Murphy et al. [35] examined whether or not planning standards were being achieved in Growth Areas. Their study found that only 26% of households in Growth Areas were within 1 km of a supermarket [35]. Further studies have also reported greater access to supermarkets in established LGAs compared to Growth Areas in Melbourne [35,36,37]. In contrast, another study found that there was greater access to fast-food outlets (e.g., takeaway franchises) in established LGAs [26], although a different study found that fast-food and independent takeaway outlets were ubiquitous across Melbourne [36]. In-store healthiness has also been shown to vary by geographic area, with shelf space in supermarkets located in urban fringe areas (i.e., Growth Areas and LGAs located in the outer ring of Melbourne) dedicating less shelf space to sales of fruit and vegetables and having more checkouts with non-alcoholic carbonated beverages than other urban areas [38].

In this paper, we aimed to explore changes in the food environment across geographic areas in metropolitan Melbourne between 2008 and 2016. We formulated the following research questions:

1. How did the density of food outlets change in Melbourne between 2008 and 2016?
2. Did the ‘healthiness’ of food outlets in Melbourne vary with distance from Central Business District; and, how has this changed over time between 2008 and 2016?
3. Did the density of different types of food outlets vary with distance from Central Business District in Melbourne; and, how has this changed over time between 2008 and 2016?

## 2. Materials and Methods

### 2.1. Study Design

A repeat cross-sectional study examining the food environment in Melbourne, Australia, over eight years at four time points (2008, 2012, 2014, and 2016) was carried out.

### 2.2. Food Outlet Data Collection and Management

Retrospective food outlet data (food outlet name, type, address) for all Melbourne LGAs (n = 31) was extracted from hard copy Yellow and White Pages business directories for 2008, 2012, 2014, and 2016. Data extracted for each calendar year were *food outlet name; address; Yellow Pages classification if applicable; and data source* (i.e., Yellow Pages or White Pages). Outlet address and suburb was matched with postcode and LGA using the Victorian Electorates by Locality and Postcode dataset [39]. 

The particular years selected align with state surveys on health outcomes [40], providing opportunities for investigating associations between the food environment and health outcomes, such as relationships with weight status and dietary intake. Yellow Pages and White pages publish government and commercial lists of businesses from information provided by telecommunications services [41]. These businesses are listed under subheadings/categories. Due to the retrospective nature of this study, ground truthing was not performed. However, limited virtual ground truthing was carried out in 2019. This involved checking whether outlets that were listed in early years of the study, but not subsequent years, were still operating. If the outlet was found to still be in operation in 2019, it was assumed that the outlet was in fact in operation until 2018, even if they were not listed as such. Virtual ground truthing was conducted in May 2019, and involved Google and Google Street View searches for the food outlets at the specified address, inspection of the retailer’s website, store front and internal photos, food offerings and menu if available. A relevant Australian food retail outlet classification tool from a validated study was utilised to identify the subheadings/categories that would be extracted [22]. Supplementary Table S1 presents the classifications and subheadings extracted from the Yellow and White Pages.

Data extraction excluded a limited number of retailers where the primary product for sale was not food, as they were unlikely to be visited on a day-to-day basis to purchase food (e.g., pharmacies and liquor stores); or, where the business listing subheading/categorisation was not consistently listed across the study period (e.g., ‘nut shop’ was listed in 2008 and 2012 but not 2014 or 2016).

Duplicate food outlets (same name, shop number and address) within a particular year were removed. Consistent with previous food environment studies, food outlets within the Central Business District (CBD; Postcode: 3000) were excluded due to outlets in this area largely servicing visitors (i.e., employees or tourists) [42].

### 2.3. Food Outlet Data Preparation

#### 2.3.1. Food Outlet Classification Tool

The Food Environment Score (FES) tool [22] was used to attribute a healthiness score to food outlets. It uses a 20-point scoring system ranging between −10 (least healthy) and +10 (most healthy) [22]. The FES is a healthiness rating system developed specifically for Australian food outlet types in Australian residential communities and was considered the most relevant tool for this study [22]. In developing the tool, twenty-six Australian public health nutrition experts took part in two rounds of a modified Delphi Survey in 2013 and 2014, with median healthiness scores from both rounds highly correlated. The FES also closely aligns with an earlier healthiness classification tool developed by Australian and international academic researchers with healthiness values attributed to food outlets falling within a close range [23]. Both healthiness rating tools have reported significant results in earlier studies examining the relationship between the healthiness of the food environment in Australia and dietary behaviors [14,23]. One of these tools also reported a strong positive correlation between the FES tool and a previously recognized scoring ratio [14]. In applying the FES tool to this study, we adapted the tool to include an additional food outlet type ‘salad bar/sushi bar’, which we assigned a healthiness score of +5. This adaptation was made to reflect relatively recent changes in Australian food environments, in consultation with a registered dietitian.

#### 2.3.2. Food Outlet Classification

Food outlets were classified into one of 17 food outlet types using the adapted FES (Supplementary Table S2) by the lead author, with cross validation of one percent (n = 491) of the sample by a registered dietitian. Store types were collapsed into seven groups based on similarity in regard to the FES definitions, food offerings and healthiness score; and finally into three groups according to healthiness score (Table 1).

### 2.4. Classification of Local Government Areas (LGAs)

**Figure 1:**
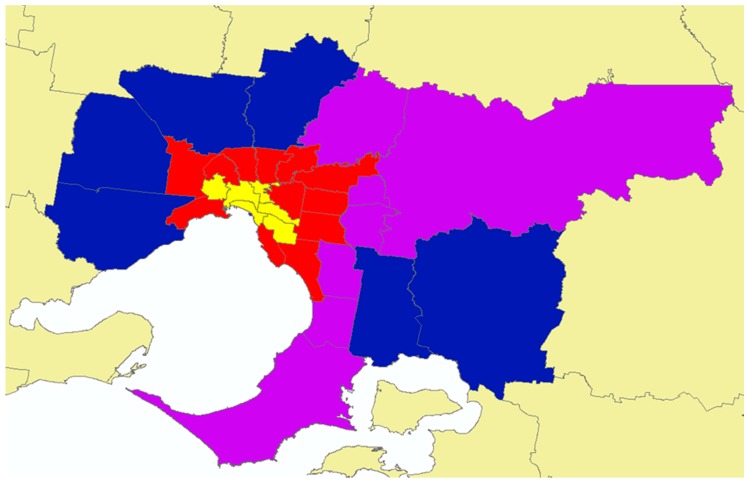
Local government areas classified by ring-like placement around Melbourne’s Central Business District (LGA-Rings). Local Government Areas: Yellow = Inner Ring; Red = Middle Ring; Purple = Outer Ring; Blue = Growth Areas.

Each of Melbourne’s 31 LGAs was classified using a method adapted from an earlier study [27] as Inner Ring (n = 6), Middle Ring (n = 12), and Outer Ring (n = 7) based on the ring-like placement of the LGAs around the CBD (Figure 1). A fourth group was defined comprising of six LGAs identified as Growth Areas (n = 6), a classification also used in earlier studies and strategic documents [35,44]. This classification will be referred to as ‘LGA-Rings’ (Supplementary Table S3 presents each LGA name within each LGA-Ring).

### 2.5. Food Environment Measures

Two previously used measures [12,20,45] were derived to describe the food environment at the LGA level: (1) density of food outlets by ‘type’ and ‘healthiness’ (number of outlets per 10,000 population) and (2) ratio of ‘unhealthy’ (or ‘less healthy’) to ‘healthy’ food outlets. Density is a measure of availability of food outlets of a given ‘type’ within the LGA, while the ratio reflects LGA resident exposure to ‘unhealthy’ (or ‘less healthy’) food outlets relative to ‘healthy’. Density of food outlets per 10,000 population was calculated for the seven food outlet ‘types’ and three ‘healthiness’ groups. Population estimates for each LGA and year were sourced from the Australian Bureau of Statistics [30]. For each LGA and year, the ratio of ‘unhealthy’ outlets (and ‘less healthy’ outlets) relative to ‘healthy’ outlets was calculated as the number of food outlets classified as ‘unhealthy’ (or ‘less healthy’) divided by the number of stores classified as ‘healthy’. 

### 2.6. Statistical Analysis

The unit of observation for this study was LGA. Linear mixed models were fitted to estimate the mean of each food environment measure across LGA-Rings and years. First, we fitted a model including LGA-Ring, time (year as a categorical variable) and the interaction LGA-Ring × time as fixed effects, and LGA as random effect (Model 1). When the interaction was non-significant the same model (Model 2) was fitted excluding the interaction term. Sidak adjusted pairwise comparisons are reported (a) within LGA-Rings between years or within year between LGA-Rings for outcomes with significant interaction (Model 1); and (b) between levels of each factor, LGA-Ring or year (Model 2). Stata version 15.0 was used for all statistical analyses [46]. 

Approval for ethics exemption was granted by the Ethics Committee from the lead author’s institution. 

## 3. Results

The population of Greater Melbourne increased by 21% from 4.02 million in 2008 to 4.85 million in 2016. Over the same period, the total number of food outlets increased by 35% from 10,777 to 13,580 and density increased by 3.1 outlets per 10,000 population from 24.9 to 28.0 (Table 2). Between 2008 and 2016, the food outlet density increased for Sushi Bars (470% growth), Major Supermarkets (226%), Restaurant Café Franchise (67%) and Fast-food Franchise (59%) and decreased for Poultry (−83.8%) and Sandwich shops (−39.5%).

Supplementary Table S4 presents the mean density of all categories of food outlet by ‘type’, ‘healthiness’ and healthiness ratios. Supplementary Table S5 presents pairwise comparisons between LGA-Rings and years for all categories of food outlet by ‘type’, ‘healthiness’ and also ratios (excluding ‘Fast-food’ and ‘Unhealthy’ food outlets).

### 3.1. Food Outlets Grouped by Healthiness: Density over Time and across LGA-Rings

**Figure 2:**
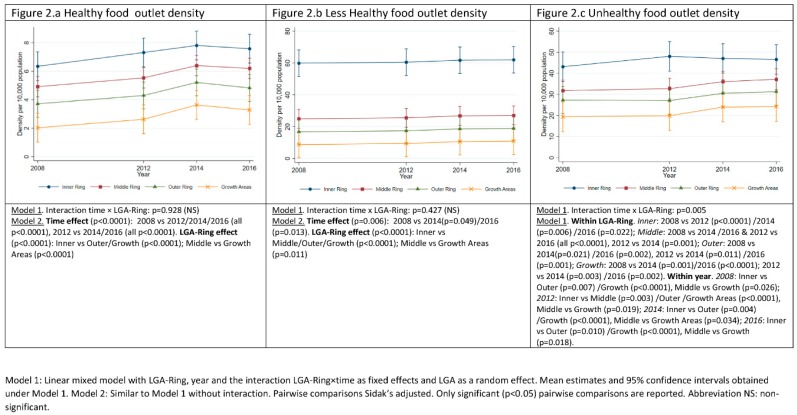
Density of healthy, less healthy and unhealthy food outlets over the period 2008–2016 across local government areas grouped by distance from Greater Melbourne Central Business District (LGA-Rings).

Figure 2 shows the estimated mean density of food outlets within each retailer ‘healthiness’ classification (see Table 1) by LGA-Ring and study year. Across all ‘healthiness’ categories, food outlet density decreased with distance from the CBD, i.e., from LGA-Ring Inner to Growth Areas. Over time both ‘unhealthy’ and ‘healthy’ food outlet density increased, whereas ‘less healthy’ food outlet density remained relatively stable. ‘Healthy’ food outlet density increased in all LGA-Rings over the study period, with no significant difference between LGA-Rings in the rate of increase. However, density of ‘healthy’ outlets was significantly different between the Inner and Middle Ring compared to Growth Areas, as was the Inner Ring compared to the Outer Ring (Figure 2a). The difference in mean ‘healthy’ retail outlet density was highest between Inner Ring and Growth Areas (4.37; 95%CI: 2.53, 6.2), and the mean density increased by 1.23 outlets per 10,000 population (95%CI: 0.94, 1.52) between 2008 and 2016 across all LGA-Rings. The difference in mean density of ‘less healthy’ retailers was greatest between Inner Ring and Growth Areas (51.12; 95%CI: 35.37, 66.87), mean density increasing by 1.23 (95%CI: 0.94, 1.52) between 2008 and 2016. Density of ‘unhealthy’ food outlets decreased at different rates with distance from CBD (Figure 2c). Mean density of ‘unhealthy’ food outlets increased between 2008 and 2012 in the Inner Ring (3.46; 95%CI: 0.32, 6.59) at which point it stabilized, the mean density in all other LGA-Rings increasing between 2012 and 2014 stabilizing between 2014 and 2016 (Figure 2c; Supplementary Table S6). The largest difference in mean density of ‘unhealthy’ retailers was between Inner Ring and Growth Areas in 2012 (28.19; 95%CI: 14.94, 41.45), and greatest growth in mean density occurred between 2008 and 2016 in the Middle Ring (5.38; 95%CI: 3.17, 7.59).

### 3.2. Ratio of Unhealthy or Less Healthy Food Outlets to Healthy Food Outlets over Time and across LGA-Rings

**Figure 3:**
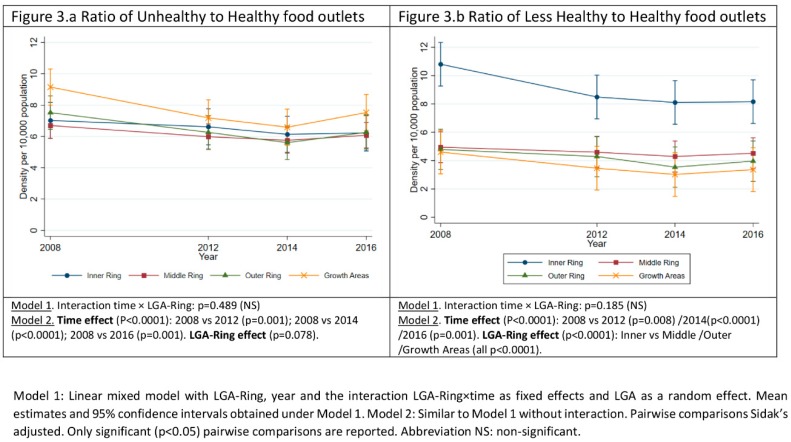
Ratio of Unhealthy and Less Healthy to Healthy food outlets over the period 2008–2016 and across local government areas grouped by distance from Greater Melbourne Central Business District (LGA-Ring).

There was no significant difference between LGA-Rings in the temporal trend for the ratio of ‘unhealthy’ and ‘less healthy’ to ‘healthy’ food outlets (Model 1) (Figure 3). The trend of decreasing density as LGA distance from CBD increased was reversed in this instance only, Growth Areas having the highest ratio of ‘unhealthy’ to ‘healthy’ food outlets although not significantly different from the other LGA-rings (Figure 3a). The ratio of ‘less healthy’ to ‘healthy’ food outlets was higher in the Inner Ring compared to all other LGA-rings, Inner Ring decreasing between 2008 and 2016 from 10.8 (95%CI: 3.6, 18.01) to 8.16 (95%CI: 5.31, 11.01) (Figure 3b).

### 3.3. Food Outlets Grouped by Store Type: Density over Time and across LGA-Rings

**Figure 4:**
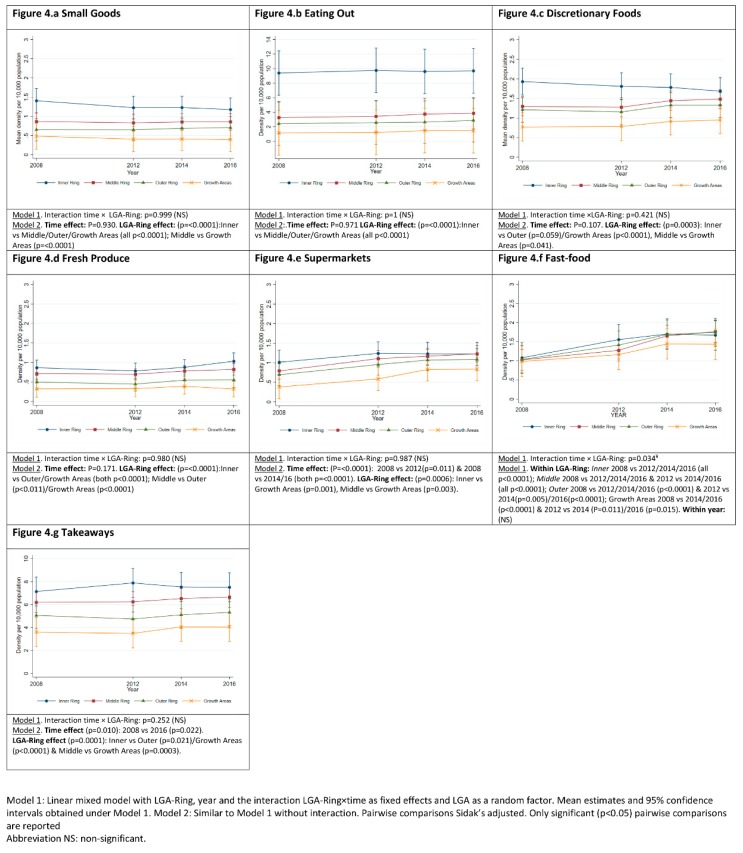
Food retail outlets grouped by type: density per 10,000 population over the period 2008-2016 and across local government areas grouped by distance from Greater Melbourne Central Business District.

Mean density of ‘Takeaways’, was consistently the food outlet ‘type’ with the highest density across all LGA-Rings (Figure 4g) ranging from a 7.89 (95%CI: 6.65, 9.26) in the Inner Ring to 3.49 (95%CI: 1.52, 5.45) in Growth Areas (both in 2012). Density of ‘Takeaways’ increased by 0.4 (95%CI: 0.04, 0.76) between 2008 and 2016. Inner Ring had significantly more ‘Takeaways’ than the Outer Ring (2.46; 95%CI: 0.25, 4.68) and Growth Areas (3.72; 95%CI: 1.42, 6.02), and Middle Ring more than Growth Areas (2.61; 95%CI: 0.62, 4.6). ‘Fast-food’ density increased in all LGA-rings over the study period (2008–2016), the Middle Ring experienced the highest growth of 73% (0.74; 95%CI: 0.57, 0.91) and Growth Areas reported the lowest growth of 28% (0.45; 95%CI: 0.2, 0.7) with no significant difference across LGA-Rings (Figure 4f; Supplementary Table S7). 

Density of ‘Supermarkets’ increased at a slower rate than ‘Takeaways’ and ‘Fast-food’ over the study period, increasing by a mean density of 0.39 (95%CI: 0.16, 0.61) outlets per 10,000 population. ‘Supermarket’ density was higher in the Inner and Middle Ring compared to Growth Areas; mean difference 0.52 (95%CI: 0.16, 0.88) and 0.41 (0.1, 0.72) respectively (Figure 4e). Between 2008 and 2016 ‘Supermarket’ density in the Middle Ring (1.23; 95%CI: 1.03, 1.43) increased to meet that of the Inner Ring (1.23; 95%CI: 0.76, 1.69), while density in the Outer (1.09; 95%CI: 0.79, 1.38) and Growth Area (0.84; 95%CI: 0.67, 1) remained 11.4% and 32% lower respectively. 

Compared to the Middle Ring, Inner Ring had a higher mean density of ‘Small Goods’ and ‘Eating out’, by 0.4 (95%CI: 0.15, 0.65) and 6.04 (95%CI: 3.52, 8.57) respectively. Compared to Outer Ring and Growth Areas, Inner Ring also had a higher mean density of ‘Small Goods’, ‘Eating Out’, ‘Discretionary Foods’ and ‘Fresh Produce’. Compared to Outer Ring and Growth Areas, Middle Ring had a higher mean density of ‘Fresh Produce’, by 0.24 (95%CI: 0.04, 0.45) and 0.42 (95%CI: 0.2, 0.63) respectively. Middle Ring also had a higher mean density of ‘Discretionary Foods’ compared to Growth Areas (0.52, 95%CI: 0.01, 1.02) (Figure 4a–d).

## 4. Discussion

As a measure food availability, density of food outlets per capita increased during the study period. We observed that new Growth Areas had fewer food outlets per capita overall relative to established LGAs and LGAs closer to the CBD. Inner Melbourne had greater availability of all food outlets (excluding ‘Fast-food’) with availability decreasing incrementally as LGA distance from CBD increased. ‘Fast-food’ was the only food outlet ‘type’ where a similar density and growth was observed over time across Melbourne. ‘Fast-food’ growth trends suggest establishment of new outlets is closely aligned with population density. In contrast, the number of ‘Supermarkets’ per capita was higher in the all other LGA-Rings compared to Growth Areas, suggesting increasing demand for ‘Supermarkets’ is not driven by population growth. As a result of these dissimilar growth patterns, Growth Areas have developed in a way that they have the highest ratio of ‘unhealthy’ food outlets to ‘healthy’ food outlets with as much as nine ‘unhealthy’ to one ‘healthy’ outlet in these areas. 

The findings from the current study are consistent with earlier studies in terms of rapid growth in access to food outlets, and trend towards a greater density of unhealthy food outlets relative to healthy food outlets. For example, an English study identified an 80% growth in food outlets overall between 1980 and 2000 with the most dramatic growth observed for takeaways and restaurants [47]. A second study reported density per 10,000 population (using data from the 2001 United Kingdom Census) of takeaway food outlets and supermarkets in Norfolk (United Kingdom) almost doubled between 1990 and 2008 by 45% and 29% respectively [45]. This was also the case in a Western Australia study examining density within 1.6 km of a residential address, the number of unhealthy outlets increasing at a faster rate in comparison to healthy outlets between 2004 and 2011 [48].

Understanding how dietary behaviours and healthy weight are influenced by food environments in which unhealthy food outlets grow at a rate outpacing that of healthy food outlets will be pertinent to future action. The FES tools have been previously used to examine healthiness of the food environment and its effect on dietary behaviours, albeit at a single time point. In the Illawara region of New South Wales (Australia), suburbs that had a higher average FES (higher being healthier) were associated with a higher consumption of fruit and vegetables [14]. Another study comparing areas using the sum of healthy outlet FES versus sum of unhealthy outlet FES, reported that a people living in urban areas of Melbourne with a higher healthy FES were more likely to report ‘never purchasing fast-food’ compared to those with less favourable healthy FES scores [23]. Informed by these findings, results from our study suggest an increase in unhealthy outlets, and an imbalance between the density of unhealthy to healthy outlets, may negatively influence dietary behaviours and in turn prevalence of overweight and obesity. Whether the relationship between the food environment and diet and obesity remains stable as the food environment changes (i.e., if obesity rates continue to increase as the food environment becomes unhealthier) will be an important relationship to understand.

A ‘food swamp’ is a spatial metaphor to describe neighbourhoods where there is a higher density of food outlets selling unhealthy quick serve foods, which are energy dense and nutrient poor, relative to the density of food outlets selling healthy options [49,50,51]. As such, we observed characteristics of a food swamp in Melbourne which parallels studies from Western Australia and Canada where ‘food swamps’ were more prevalent than ‘food deserts’ [43,46]. This was also the case in deprived areas in New Zealand, which had a higher density of unhealthy food outlets (fast-food, takeaway, convenience stores) relative to healthy food outlets (supermarkets, fruit and vegetable stores) compared to the least deprived areas [20]. 

Evidence is emerging that this imbalance in food retail mix has health implications. A study from the United States of America identified food swamps as being greater predictors of adult obesity than food deserts (i.e., areas with limited access to healthy food) [51]; and one Australian study found when examining the ratio of healthy (i.e., supermarkets and green grocers) to unhealthy (i.e., fast-food outlets) that a higher BMI was associated with higher density of unhealthy outlets (25% or more of total outlets located within 1.6 km and 3.2 km from home) [12]. Despite non-significance, the higher ratio of unhealthy to healthy outlets in Melbourne’s Growth Areas should raise concerns given the reported higher prevalence of overweight and obesity compared to established suburbs [26,40]. Similar patterns were observed in Perth (Western Australia), where despite improving over time, the proportion of healthy outlets to unhealthy outlets in new developments were consistently lower than established areas [48].

Many factors such as lower housing density, increasing commuting distance to workplaces, car dependence, poor public transport, cultural factors and increased distances to open space and food retail may contribute to the relationship between the food environment and obesity [26,52]. Nevertheless, disparities in Melbourne’s food environments have the potential to disadvantage communities in Growth Areas due to limited availability and exposure to food resources, which in turn may result in greater reliance on vehicle access and car travel to obtain healthy food [26,48,53]. 

### 4.1. Strengths

To date, no known Australian or international study has examined density, type or ratio of food outlets in a large metropolitan city over time. Examining the food environment in a larger geographic unit than earlier studies provides for a more comprehensive understanding of food availability at a population level, compared to studies examining only food environments immediately surrounding a residential home. Several steps were taken to improve the quality of food outlet data in this study: data were sourced from multiple sources (i.e., Yellow Pages and White Pages); and rigorous cross-checking (i.e., Google search, virtual ground truthing) were undertaken to increase completeness [54]. By taking a holistic approach and encapsulating all food outlet businesses, rather than only examining large global chains (absolute measures), this study provides the most comprehensive snapshot of food outlet trends in Australia.

### 4.2. Limitations

The assessment of food outlet healthiness was based on a descriptive classification of food outlet type. Accordingly, it was generally not possible to take into account the types of products available for sale and other aspects of the in-store environment that may impact the healthiness of purchases from each outlet. While in-store assessments (e.g., using the NEMS-R tool) may have given a more accurate measure of food outlet healthiness [55], it was not feasible in this study given the temporal nature, large-scale and resource constraints. The FES provides only a generalized healthiness measure of food outlet types which may not reflect in-store variation in healthiness of food offerings and how this changes over time. Additionally, the FES may not identify the emergence of new food outlet types over time. Further development, refinement, and continued updating of the FES will create a more nuanced tool that can be used at scale (i.e., across metropolitan, regional, rural and remote areas). Another key limitation of this study is that the datasets used may not represent all food outlets available, potentially introducing bias in density estimates [54]. For example, some food outlet types were not listed in the Yellow or White Pages (e.g., farmers markets, local produce stalls, wholesalers/food cooperatives and service stations convenience stores), meaning they were unable to be included within this study. Therefore, estimates of food outlet density in this study are likely to be lower than the true density of food retail. Additionally, due to the retrospective nature of this study, virtual ground truthing could only be performed in 2019, limiting the ability to confirm store operation or closure at each study time point. Nevertheless, virtual ground truthing was important to identify food outlets that were still in operation but may have ceased using fixed-line phone services, and thus would not have been listed in the Yellow Pages. It is also possible that some food outlets were never listed in the Yellow Pages and as such would not have been identified. Additionally, alternate food sources, such as online purchasing for home delivery are likely to be under-represented also, because online food retailers may not have a physical food retail address listed in the Yellow or White Pages. Additionally, food outlet businesses listed do not specify that they provide a food delivery service, nor the geographical area in which they service which could be larger than the single LGA in which their physical premises is located. This study did not take into account the geographical differences in the size of the LGAs, and therefore does not elucidate spatial disparities in food outlet access which would be useful. Lastly, this study did not examine associations between the food environment and measures of dietary intake or obesity prevalence, which is an important avenue for exploration in future studies. 

### 4.3. Implications for Practice

This study identifies disparities in healthiness of food environments whereby Growth Areas, where obesity prevalence is highest, appear to have experienced growth in ‘unhealthy’ food outlets, increased exposure to ‘fast-food’ and subsequent risk of obesity [56]. Development and implementation of planning policies to limit availability of unhealthy food outlets, and increase healthy food outlet availability and accessibility are urgently needed across all areas, and particularly for Growth Areas [5,12]. One mechanism could be the inclusion of ‘public health considerations’ within urban planning legislative frameworks to provide LGAs the authority to develop and implement healthy food environment initiatives through urban planning [26]. 

In our study, an increasing density of supermarkets was observed and it is known that supermarkets sell unhealthy foods and beverages and encourage purchasing through strategic placement and price promotions of these products [38,57,58,59]. Since Australians make two-thirds of food and beverage purchases (excluding alcohol) at supermarkets [60], further examination of the implications of in-store promotion and placement is needed.

Additionally, policies that facilitate a healthier food environment (e.g., more fruit and greengrocers, butchers, fish and poultry shops) in Growth Areas represent a clear strategy achievable through LGAs. Policies could include support for independent fresh food outlet establishment (e.g., through reduced council rates). Or, subsidised mobile greengrocers that can visit areas with limited access to healthy food and high food insecurity such as the ‘Community Grocer’ program, which is led by the community with support from the City of Melbourne [61]. 

This study highlights the importance of routine food environment monitoring, which helps identify widening disparities within communities and cities. Temporal data are crucial to understanding trends in disparities in environments, particularly given the dynamic growth in population in the most affected areas. Better understanding mechanisms by which rapid urban growth and development of expansive areas with low-density housing results in higher obesity prevalence is also needed to underpin prevention efforts [52] and support policy development. Use of Geographic Information Systems to examine food outlet access would improve understanding of food outlet accessibility. Studies examining the feasibility of increasing housing density and land use mix in Growth Areas would also be valuable to inform strategic planning for healthy urban design to support public health. 

## 5. Conclusions

This study provides strong evidence on the proliferation of food outlets and increasing food availability in Melbourne, and the disproportionate dominance of ‘unhealthy’ food outlets relative to ‘healthy’ outlets across Melbourne. Evidence indicates that an inequitable balance in availability and exposure to food resources may exist, particularly with regard to the limited availability of healthy food outlets in contrast to unhealthy outlets in Melbourne’s Growth Areas. Large-scale food environment policy change and well-managed population growth will be important components of efforts to improve population diets and address obesity.

## Figures and Tables

**Table 1:** Food outlet classifications.

Food Outlets Grouped by ‘Type’		Food Outlets Grouped by ‘Healthiness’ Score
1. Supermarkets: Minor and Major Supermarkets	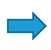	1. Healthy (FES range: +5 to +10): Supermarkets, Fruit and greengrocer, Butcher, Fish, Poultry shop, Salad/Sandwich/Sushi bar.
2. Fresh Produce: Fruit and greengrocer, Butcher, Fish, Poultry shop		
3. Eating Out: Cafes and Restaurants (Independent and Franchise), and Pubs		2. Less Healthy (FES range: −4 to +4): Cafes and Restaurants (Independent and Franchise), Bakers, Delis.
4. Small Goods: Bakers, Delis, Sandwich and Sushi		
5. Fast-food: Takeaway Franchise		3. Unhealthy (FES range: −10 to −5): Fast-food, Takeaway independent, Pubs, General stores and Specialty extra.
6. Takeaways: Takeaway Independent		
7. Discretionary Foods *: General stores and Specialty extra		

FES: Food Environment Score reflects the perceived healthiness value allocated to particular food outlet types. Range: −10 to +10. * Discretionary Foods are described in the Australian Dietary Guidelines as foods and drinks “not necessary for a healthy diet and are too high in saturated fat and/or added sugars, added salt or alcohol or low in fibre” [43].

**Table 2:** Annual resident population estimates, number of food outlets and average density per 10,000 population across Greater Melbourne’s 31 local government areas from 2008 to 2016.

Measures	Year				
	2008	2012	2014	2016	% Change ^#^
**Greater Melbourne population**	4,020,094	4,356,752	4,566,216	4,851,674	20.7
**Food outlet classification (healthiness value):**	**Number of outlets (density per 10,000 population)**				
Total	10,077 (25)	11,415 (26.2)	12,943 (28.4)	13,580 (28)	+34.8(11.7)
Baker (0)	804 (2)	841 (1.93)	879 (1.93)	911 (1.88)	+13 (−6.11)
Butcher (+9)	386 (0.96)	422 (0.97)	489 (1.07)	502 (1.03)	+30 (7.76)
General Stores (−5)	532 (1.32)	523 (1.2)	664 (1.45)	687 (1.42)	+29 (7)
Delicatessen (0)	297 (0.74)	262 (0.6)	255 (0.56)	253 (0.52)	−15 (−29.42)
Fruit & Greengrocer (+10)	304 (0.76)	278 (0.64)	330 (0.72)	337 (0.69)	+11 (−8.15)
Fish Shop (+9)	110 (0.27)	118 (0.27)	177 (0.39)	181 (0.37)	+65 (36.34)
Major Supermarkets (+5)	99 (0.25)	309 (0.71)	394 (0.86)	390 (0.8)	+294 (226.42)
Minor Supermarkets (+5)	440 (1.09)	508 (1.17)	552 (1.21)	639 (1.32)	+45 (20.34)
Poultry Shop (+9)	97 (0.24)	90 (0.21)	128 (0.28)	19 (0.04)	−80 (−83.77)
Pubs (−5)	290 (0.72)	339 (0.78)	323 (0.71)	357 (0.74)	+23.1 (2)
Restaurant Café Franchise (0)	98 (0.24)	152 (0.35)	247 (0.54)	198 (0.41)	+102 (67.41)
Restaurant Café Independent (0)	3468 (8.63)	3910 (8.97)	4317 (9.45)	4672 (9.63)	+35 (11.63)
Sandwich shop (+5)	78 (0.19)	48 (0.11)	58 (0.13)	57 (0.12)	−27 (−39.45)
Specialty Core (+5)	106 (0.26)	149 (0.34)	230 (0.5)	245 (0.5)	+131 (91.52)
Specialty Extra (−8)	427 (1.06)	486 (1.12)	520 (1.14)	550 (1.13)	+29 (6.73)
Sushi bar (+5)	17 (0.04)	112 (0.26)	136 (0.3)	117 (0.24)	+588 (470.27)
Fast-food Franchise (−10)	405 (1.01)	560 (1.29)	720 (1.58)	779 (1.61)	+92 (59.38)
Takeaway Independent (−8)	2119 (5.27)	2308 (5.3)	2524 (5.53)	2686 (5.54)	+27 (5.03)

# % change 2008 to 2016.

## Supplementary Materials

The following are available online at https://www.mdpi.com/1660-4601/17/4/1321/s1, Table S1: Classifications extracted from Yellow and White Pages, Table S2: Food outlet descriptions and scores, Table S3: Local government area classification by distance from Central Business District in Melbourne, Australia, Table S4: Density of food outlet types and food outlet healthiness groups (Healthy, Less Healthy, Unhealthy) and ratio of Unhealthy, Less Healthy to Healthy food outlets across geographical areas in Greater Melbourne, Table S5: Pairwise comparisons for liner mixed model without interaction for food outlets types, food outlets grouped by healthiness and ratio of Healthy and Less Healthy to Healthy outlet over time and across local government area rings, Melbourne, Australia, 2008–2016, Table S6: Pairwise comparisons for liner mixed model with interaction for Unhealthy food outlets over time across local government area rings, Melbourne, Australia, 2008–2016, Table S7: Pairwise comparisons for liner mixed model with interaction for Fast-food outlets over time across local government area rings, Melbourne, Australia, 2008–2016.
