# Supplementary material for: Food Retail Environments in Greater Melbourne 2008–2016: Longitudinal Analysis of Intra-City Variation in Density and Healthiness of Food Outlets
Source: Int J Environ Res Public Health. 2020 Feb 19;17(4):1321. doi: 10.3390/ijerph17041321 (PMC7068484; doi:10.3390/ijerph17041321)
Supplement: Supplementary file 1 [file ijerph-17-01321-s001.pdf]

Supplementary Table 1. Classifications extracted from Yellow and White Pages

Yellow Pages Directory Classifications	White Pages - Business Directory*
Bakers	ALDI
Butchers retail	Coffee Club
Cafes	Coles Express
Cakes & pastry shops	Domino's
Chocolate & Cocoa	Gloria Jeans
Confectionary retail	Hungry Jacks
Delicatessens	Kmart
Fish & seafood retail	McDonald's
Food & /or general stores	NQR
Fruiterers & greengrocers	Safeway/Woolworths
Halal products	Target
Health foods & products	The Reject Shop
Hotels-pubs	Wendy's
Ice-cream retail	
Kosher products	
Organic products	
Restaurants	
Pizza	
Poultry retail	
Supermarkets & grocery stores	
Take away foods	

*Food outlets with subheadings were identified and extracted from the White Pages

Supplementary Table 2: Food outlet descriptions and healthiness scores

Food outlet type	Description	Health Score
Fruiterer & greengrocer	Mainly engaged in the sale of fresh fruit and vegetables; including wholesale stores with direct to public sales	10
Fish shop	Mainly engaged in the sale of fresh seafood; including wholesale stores with direct to public sales and takeaway stores also providing a range of fresh seafood.	9
Poultry shop	Mainly engaged in the sale of fresh poultry; including wholesale stores and with direct to public sales.	9
Butchery	Mainly engaged in the sale of fresh meat; including wholesale stores with direct to public sales.	9
Major Supermarket	Mainly engaged in the sale of groceries (fresh foods, canned and packaged foods, dry goods) of non-specialised (conventional) food lines. May contain a butcher or baker. Usually have 5 or more checkouts and a floor area over 1000 square meters. I.e. Woolworths, Coles, BI-LO, Franklins (no frills), ALDI	5
Minor Supermarket	Mainly engaged in the sale of groceries (fresh foods, canned and packaged food, dry goods) of non-specialised (conventional) food lines. Usually have 4 or fewer checkouts and a floor area under 1000 square meters. E.g. Independent grocer or supermarket.	5
Specialty food stores – core foods	Mainly engaged in the sale of a limited line of specialised food such as a particular gourmet food that can be defined under core food.	5
Restaurant/café – franchise	E.g. franchise restaurants and cafes; mainly engaged in the preparation and sale of meals/snacks for consumption on the premises; table service provided; may sell alcohol with food; may provide takeaway services.	0
Restaurant/café – local independent	e.g. restaurant in a golf club, culture-based restaurant/café which is not a take-away such as Mexican, Thai, Chinese etc. ; mainly engaged in the preparation and sale of meals/snacks for consumption on the premises; table service provided; may also sell alcohol with food, may provide takeaway services.	0
Sandwich shop	Mainly engaged in the preparation of filled bread products like sandwiches or rolls.	5
Salad/sushi bar	Mainly engaged in the preparation of salads and sushi.	5
Delicatessen	Mainly engaged in the sale of specialty packaged or fresh products such as cured meats and sausage, pickled vegetables, dips, bread and olives; may also provide dine in meals.	0
Bakery	Mainly oriented towards bread, biscuits, pastries or other flour products with or without packaging.	0
General store	Mainly engaged in the sale of a limited line of groceries generally includes milk, bread and canned and packaged foods.	-5
Specialty food store – extra foods	Mainly engaged in the sale of foods such as ice-creams, donuts, waffles, cakes etc. than can be defined under extra food.	-8
Pub	e.g. pub within a bowing park, pub inside a private gambling club; food primarily engaged in selling alcoholic beverages where consumers can order and consume the alcoholic drinks in premises; can also be part of park or private club.	-5
Take-away local independent	E.g. kebab, fish & chips, burger, chicken shops, local pizza, mainly engaged in the preparation and sale of meals/snacks that are ready for immediate consumption; table service not provided; meals can be eaten on site; taken away or delivered; shop is not a franchise.	-8

Take-away franchise store	E.g. McDonalds, KFC, Subway; mainly engaged in the preparation and sale of meals (excludes donuts, drinks, ice-cream etc.)/snacks that area ready for immediate consumption; table service not provided; meal can be eaten on site, taken away or delivered; the food shop is a franchise/chain store with food being sold in specialised packaging.	-10

Source: adapted from **Moayyed et al. 2017**[(22)]

Supplementary Table 3. Local government area classification by distance from the Central Business District in Melbourne, Australia

Local government area classification by distance to CBD		
**LGA-Ring classification**	Approximate distance from CBD	Local Government Area [#]
**Inner**	<15km	Glen Eira, Maribyrnong, Melbourne (excluding CBD), Port Phillip, Stonington, Yarra
**Middle**	15- 25km	Banyule, Bayside, Boroondara, Brimbank, Darebin, Hobsons Bay, Kingston, Manningham, Monash, Moonee Valley, Moreland, Whitehorse
**Outer**	25-55km	Frankston, Dandenong, Knox, Maroondah, Manningham, Nillumbik, Yarra Ranges
**Growth Areas[#]**	30-70km	Casey, Cardinia, Hume, Melton, Whittlesea, Wyndham

Acronym: CBD: Central Business District, Growth Areas:

Victorian Growth Areas are local government areas designated by state government to house new population growth

Supplementary Table 4. Density of food outlets types and food outlet healthiness groups (Healthy, Less Healthy, Unhealthy) and ratio of Unhealthy, Less Healthy to Healthy food outlets across geographical areas in Greater Melbourne, Australia, 2008-2016

		Year			
		2008	2012	2014	2016
Food outlet group/type	LGA-Ring	Mean (95%CI)	Mean (95%CI)	Mean (95%CI)	Mean (95%CI)
Density Healthy food outlets	Inner	6.34(4.2,8.48)	7.31(5.93,8.69)	7.8(5.77,9.83)	7.58(5.69,9.47)
	Middle	4.91(4.29,5.54)	5.53(5.08,5.98)	6.39(5.75,7.04)	6.19(5.61,6.78)
	Outer	3.71(2.2,5.21)	4.29(2.65,5.94)	5.21(3.42,7)	4.82(3.1,6.55)
	Growth Areas	2.03(1.17,2.88)	2.62(1.48,3.77)	3.63(2.64,4.62)	3.28(2.26,4.3)
Density Less Healthy food outlets	Inner	61.66(33.82,89.51)	61.22(36.45,86)	60.82(37.46,84.19)	60.56(38.17,82.96)
	Middle	24.08(19.98,28.18)	25.27(21.63,28.9)	27.19(23.35,31.03)	27.89(23.77,32.01)
	Outer	16.49(10.93,22.05)	17.6(11.75,23.44)	18.56(12.48,24.65)	19.08(12.95,25.22)
	Growth Areas	9.15(5.42,12.88)	9.18(4.79,13.57)	10.86(7.71,14)	10.61(7.51,13.71)
Density Unhealthy food outlets	Inner	43.14(28.37,57.9)	48.07(36.6,59.53)	47.05(35.52,58.58)	46.59(34.79,58.4)
	Middle	31.79(28.29,35.28)	32.73(29.38,36.07)	36.03(32.4,39.67)	37.17(32.94,41.4)
	Outer	27.3(16.4,38.2)	27.08(16.29,37.87)	30.51(18.07,42.96)	31.28(18.65,43.9)
	Growth Areas	19.36(10.15,28.58)	19.88(9.74,30.02)	23.98(16.78,31.19)	24.23(17.74,30.72)
Ratio Unhealthy to Healthy food outlets	Inner	7.02(4.89,9.15)	6.61(5.32,7.9)	6.13(5.01,7.25)	6.22(5.09,7.35)
	Middle	6.69(5.6,7.79)	5.98(5.27,6.69)	5.75(4.99,6.51)	6.07(5.34,6.81)
	Outer	7.52(6.5,8.54)	6.26(5.18,7.34)	5.6(4.02,7.18)	6.26(4.39,8.13)
	Growth Areas	9.15(5.31,12.99)	7.18(5.66,8.71)	6.59(5.79,7.39)	7.52(6.74,8.31)
Ratio Less Healthy to Healthy food outlets	Inner	10.8(3.6,18.01)	8.49(4.77,12.22)	8.11(4.62,11.59)	8.16(5.31,11.01)
	Middle	4.95(4.2,5.69)	4.59(3.91,5.28)	4.3(3.68,4.92)	4.52(3.89,5.15)
	Outer	4.79(3.89,5.69)	4.29(3.68,4.9)	3.54(3,4.09)	3.96(3.36,4.57)
	Growth Areas	4.6(4.03,5.18)	3.46(3.11,3.81)	3.02(2.46,3.58)	3.36(2.39,4.34)
Small Goods	Inner	1.41(0.88,1.94)	1.23(0.85,1.61)	1.23(0.84,1.62)	1.18(0.82,1.53)
	Middle	0.86(0.61,1.12)	0.83(0.59,1.08)	0.85(0.62,1.08)	0.86(0.62,1.09)
	Outer	0.65(0.38,0.93)	0.65(0.38,0.92)	0.69(0.44,0.94)	0.7(0.44,0.97)
	Growth Areas	0.48(0.24,0.73)	0.41(0.21,0.6)	0.41(0.23,0.59)	0.39(0.21,0.57)

**Fresh Produce**	**Inner**	0.86(0.62,1.11)	0.79(0.57,1.01)	0.88(0.64,1.11)	1.02(0.78,1.26)
	**Middle**	0.72(0.56,0.87)	0.7(0.56,0.84)	0.78(0.63,0.93)	0.82(0.66,0.98)
	**Outer**	0.5(0.35,0.66)	0.45(0.31,0.59)	0.56(0.39,0.72)	0.55(0.36,0.75)
	**Growth Areas**	0.34(0.23,0.44)	0.34(0.22,0.45)	0.39(0.28,0.51)	0.32(0.2,0.44)
**Discretionary Foods**	**Inner**	1.93(1.51,2.35)	1.81(1.49,2.13)	1.78(1.32,2.24)	1.69(1.29,2.08)
	**Middle**	1.3(1.16,1.43)	1.28(1.11,1.44)	1.44(1.31,1.58)	1.48(1.3,1.67)
	**Outer**	1.21(0.87,1.55)	1.16(0.78,1.53)	1.33(0.92,1.73)	1.33(0.95,1.71)
	**Growth Areas**	0.87(0.58,1.15)	0.88(0.69,1.06)	0.91(0.62,1.2)	0.95(0.69,1.21)
**Supermarkets**	**Inner**	1.03(0.51,1.54)	1.24(0.78,1.7)	1.23(0.78,1.68)	1.23(0.76,1.69)
	**Middle**	0.79(0.54,1.04)	1.11(0.93,1.29)	1.17(0.99,1.34)	1.23(1.03,1.43)
	**Outer**	0.72(0.38,1.05)	0.96(0.67,1.24)	1.07(0.8,1.34)	1.09(0.79,1.38)
	**Growth Areas**	0.39(0.25,0.53)	0.59(0.43,0.75)	0.83(0.73,0.93)	0.84(0.67,1)
**Eating Out**	**Inner**	9.4(2.56,16.24)	9.75(3.11,16.4)	9.61(3.11,16.11)	9.7(3.16,16.24)
	**Middle**	3.25(1.77,4.74)	3.44(1.93,4.95)	3.74(2.11,5.36)	3.86(2.14,5.59)
	**Outer**	2.41(1.02,3.81)	2.57(1.07,4.07)	2.64(1.12,4.16)	2.87(1.2,4.55)
	**Growth Areas**	1.16(0.48,1.84)	1.21(0.46,1.96)	1.46(0.69,2.22)	1.45(0.64,2.26)
**Fast-food**	**Inner**	1.08(0.67,1.49)	1.56(1.03,2.08)	1.7(1.18,2.22)	1.67(1.22,2.12)
	**Middle**	1.02(0.77,1.28)	1.28(1.02,1.53)	1.65(1.34,1.97)	1.76(1.42,2.11)
	**Outer**	1.04(0.55,1.52)	1.41(0.81,2.01)	1.69(0.93,2.45)	1.74(0.96,2.53)
	**Growth Areas**	1.12(0.78,1.47)	1.16(0.61,1.72)	1.44(1.01,1.87)	1.43(0.99,1.88)
**Takeaways**	**Inner**	7.14(4.69,9.59)	7.89(6.51,9.26)	7.54(5.97,9.11)	7.51(5.99,9.03)
	**Middle**	6.2(5.28,7.12)	6.25(5.51,6.98)	6.52(5.66,7.38)	6.66(5.77,7.55)
	**Outer**	5.06(2.76,7.35)	4.75(2.78,6.72)	5.11(2.97,7.25)	5.33(3.15,7.51)
	**Growth Areas**	3.6(1.8,5.4)	3.49(1.52,5.45)	4.06(2.93,5.19)	4.05(3.15,4.95)

Supplementary Table 5. Pairwise comparisons for liner mixed model without interaction for food outlets types, food outlets grouped by healthiness and ratio of Healthy and Less Healthy to Healthy outlet over time and across local government area rings, Melbourne, Australia, 2008-2016

	Density Healthy	Density Less Healthy	Ratio Unhealthy to Healthy	Ratio Less Healthy to Healthy	Small Goods	Eating Out	Discretionary Foods	Fresh Produce	Supermarkets	Takeaways
	Contrast (95%CI)	Contrast (95%CI)	Contrast (95%CI)	Contrast (95%CI)	Contrast (95%CI)	Contrast (95%CI)	Contrast (95%CI)	Contrast (95%CI)	Contrast (95%CI)	Contrast (95%CI)
Within LGA-Ring mean density of food outlet: linear mixed model without interaction pairwise comparisons Sidak's adjusted										
Middle vs Inner	-1.5(-3.09,0.09)	**-34.96(-48.6,-21.32)**	-0.37(-1.89,1.15)	**-4.3(-6.53,-2.07)**	**-0.4(-0.65,-0.15)**	**-6.04(-8.57,-3.52)**	-0.43(-0.93,0.07)	-0.13(-0.35,0.08)	-0.1(-0.41,0.21)	-1.11(-3.1,0.88)
Outer vs Inner	**-2.75(-4.52,-0.98)**	**-43.14(-58.31,-27.96)**	-0.09(-1.78,1.6)	**-4.75(-7.23,-2.27)**	**-0.58(-0.86,-0.3)**	**-6.99(-9.86,-4.13)**	**-0.55(-1.1,0.01)**	**-0.37(-0.61,-0.13)**	-0.22(-0.57,0.12)	**-2.46(-4.68,-0.25)**
Growth Areas vs Inner	**-4.37(-6.2,-2.53)**	**-51.12(-66.87,-35.37)**	1.12(-0.64,2.87)	**-5.28(-7.85,-2.71)**	**-0.84(-1.13,-0.54)**	**-8.3(-11.21,-5.39)**	**-0.94(-1.53,-0.36)**	**-0.55(-0.8,-0.3)**	**-0.52(-0.88,-0.16)**	**-3.72(-6.02,-1.42)**
Outer vs Middle	-1.25(-2.76,0.26)	-8.17(-21.15,4.8)	0.28(-1.16,1.73)	-0.44(-2.56,1.68)	-0.18(-0.42,0.07)	-0.95(-3.42,1.52)	-0.12(-0.6,0.36)	**-0.24(-0.45,-0.04)**	-0.12(-0.42,0.17)	-1.35(-3.24,0.55)
Growth Areas vs Middle	**-2.87(-4.46,-1.28)**	**-16.16(-29.8,-2.52)**	1.49(-0.03,3.01)	-0.98(-3.2,1.25)	**-0.43(-0.69,-0.17)**	-2.25(-4.78,0.27)	**-0.52(-1.02,-0.01)**	**-0.42(-0.63,-0.2)**	**-0.41(-0.72,-0.1)**	**-2.61(-4.6,-0.62)**
Growth Areas vs Middle	-1.62(-3.39,0.15)	-7.98(-23.16,7.19)	1.2(-0.49,2.89)	-0.53(-3.01,1.95)	-0.25(-0.54,0.04)	-1.3(-4.17,1.56)	-0.4(-0.96,0.16)	-0.17(-0.42,0.07)	-0.29(-0.64,0.05)	-1.26(-3.48,0.95)

Within Year mean density of food outlet: linear mixed model without interaction pairwise comparisons Sidak’s adjusted										
2012 vs 2008	**0.67(0.38,0.96)**	0.63(-1.2,2.46)	**-1.02(-1.71,-0.33)**	**-0.92(-1.67,-0.16)**	-0.06(-0.33,0.2)	0.18(-2.41,2.78)	-0.04(-0.21,0.12)	-0.03(-0.18,0.11)	**0.26(0.04,0.49)**	0.07(-0.29,0.43)
2014 vs 2008	**1.5(1.22,1.79)**	**1.84(0.01,3.67)**	**-1.47(-2.16,-0.78)**	**-1.36(-2.12,-0.61)**	-0.04(-0.31,0.22)	0.34(-2.25,2.93)	0.08(-0.08,0.24)	0.05(-0.09,0.2)	**0.36(0.14,0.58)**	0.3(-0.06,0.67)
2016 vs 2008	**1.23(0.94,1.52)**	**2.13(0.3,3.96)**	**-0.99(-1.69,-0.3)**	**-1.1(-1.86,-0.35)**	-0.05(-0.32,0.21)	0.45(-2.14,3.05)	0.08(-0.08,0.24)	0.08(-0.07,0.23)	**0.39(0.16,0.61)**	**0.4(0.04,0.76)**
2014 vs 2012	**0.83(0.54,1.12)**	1.21(-0.62,3.04)	-0.45(-1.14,0.24)	-0.44(-1.2,0.31)	0.02(-0.24,0.27)	0.15(-2.43,2.74)	0.12(-0.04,0.28)	0.09(-0.06,0.23)	0.09(-0.13,0.31)	0.23(-0.13,0.59)
2016 vs 2012	**0.56(0.27,0.85)**	1.5(-0.33,3.33)	0.03(-0.67,0.72)	-0.19(-0.94,0.57)	0.01(-0.25,0.27)	0.27(-2.32,2.86)	0.13(-0.04,0.29)	0.12(-0.04,0.27)	0.12(-0.1,0.34)	0.33(-0.03,0.69)
2016 vs 2014	-0.28(-0.57,0.01)	0.29(-1.54,2.12)	0.47(-0.22,1.17)	0.26(-0.5,1.01)	-0.01(-0.27,0.25)	0.11(-2.47,2.7)	0(-0.16,0.16)	0.03(-0.12,0.18)	0.03(-0.19,0.25)	0.1(-0.27,0.46)

Bold = results significant p≤0.05

Supplementary Table 6. Pairwise comparisons for liner mixed model with interaction for Unhealthy food outlets over time across local government area rings, Melbourne, Australia, 2008-2016

Within Year, LGA-Ring comparison				Within LGA-Ring, Year comparison			
Year	LGA-RING comparison	Contrast (95% CI)	P value	LGA-Ring	Year comparison	Contrast (95% CI)	P Value
2008	Middle vs Inner	-11.35(-22.83,0.13)	0.054	Inner	2012 vs 2008	4.93(1.8,8.06)	0.000
	Outer vs Inner	-15.84(-28.61,-3.06)	0.007		2014 vs 2008	3.91(0.78,7.04)	0.006
	Growth Areas vs Inner	-23.77(-37.03,-10.52)	0.000		2016 vs 2008	3.46(0.32,6.59)	0.022
	Outer vs Middle	-4.49(-15.4,6.43)	0.860		2014 vs 2012	-1.02(-4.15,2.11)	0.950
	Growth Areas vs Middle	-12.42(-23.9,-0.94)	0.026		2016 vs 2012	-1.47(-4.61,1.66)	0.767
	Growth Areas vs Outer	-7.94(-20.71,4.84)	0.476		2016 vs 2014	-0.46(-3.59,2.68)	0.999
2012	Middle vs Inner	-15.34(-26.82,-3.86)	0.003	Middle	2012 vs 2008	0.94(-1.27,3.15)	0.841
	Outer vs Inner	-20.99(-33.76,-8.21)	0.000		2014 vs 2008	4.24(2.03,6.46)	0.000
	Growth Areas vs Inner	-28.19(-41.45,-14.94)	0.000		2016 vs 2008	5.38(3.17,7.59)	0.000
	Outer vs Middle	-5.65(-16.57,5.27)	0.682		2014 vs 2012	3.3(1.09,5.52)	0.001
	Growth Areas vs Middle	-12.85(-24.33,-1.37)	0.019		2016 vs 2012	4.44(2.23,6.65)	0.000
	Growth Areas vs Outer	-7.2(-19.98,5.57)	0.589		2016 vs 2014	1.14(-1.08,3.35)	0.689
2014	Middle vs Inner	-11.02(-22.5,0.46)	0.067	Outer	2012 vs 2008	-0.22(-3.12,2.68)	1.000
	Outer vs Inner	-16.53(-29.31,-3.76)	0.004		2014 vs 2008	3.21(0.31,6.11)	0.021
	Growth Areas vs Inner	-23.06(-36.32,-9.81)	0.000		2016 vs 2008	3.97(1.07,6.87)	0.002
	Outer vs Middle	-5.52(-16.43,5.4)	0.704		2014 vs 2012	3.43(0.53,6.33)	0.011

	Growth Areas vs Middle	-12.05(-23.53,-0.57)	0.034		2016 vs 2012	4.2(1.3,7.09)	0.001
	Growth Areas vs Outer	-6.53(-19.3,6.24)	0.693		2016 vs 2014	0.76(-2.14,3.66)	0.982
	Middle vs Inner	-9.43(-20.9,2.05)	0.171		2012 vs 2008	0.51(-2.62,3.64)	0.999
	Outer vs Inner	-15.32(-28.09,-2.55)	0.010		2014 vs 2008	4.62(1.49,7.75)	0.001
	Growth Areas vs Inner	-22.37(-35.62,-9.11)	0.000		2016 vs 2008	4.86(1.73,7.99)	0.000
	Outer vs Middle	-5.89(-16.81,5.03)	0.638		2014 vs 2012	4.11(0.98,7.24)	0.003
2016	Growth Areas vs Middle	-12.94(-24.42,-1.46)	0.018	Growth	2016 vs 2012	4.35(1.22,7.48)	0.002
	Growth Areas vs Outer	-7.05(-19.82,5.72)	0.613		2016 vs 2014	0.24(-2.89,3.37)	1.000

Supplementary Table 7. Pairwise comparisons for liner mixed model with interaction for Fast-food outlets over time across local government area rings, Melbourne, Australia, 2008-201.6

Within Year, LGA-Ring comparison				Within LGA-Ring, Year comparison			
Year	LGA_RING comparison	Contrast (95% CI)	P value	LGA-Ring	Year comparison	Contrast (95% CI)	P value
2008	Middle vs Inner	-0.06(-0.71,0.59)	1.000	Inner	2012 vs 2008	0.48(0.24,0.71)	0.000
	Outer vs Inner	-0.04(-0.77,0.68)	1.000		2014 vs 2008	0.62(0.39,0.86)	0.000
	Growth Areas vs Inner	-0.1(-0.85,0.66)	1.000		2016 vs 2008	0.59(0.36,0.83)	0.000
	Outer vs Middle	0.01(-0.61,0.63)	1.000		2014 vs 2012	0.14(-0.09,0.38)	0.484
	Growth Areas vs Middle	-0.04(-0.69,0.62)	1.000		2016 vs 2012	0.12(-0.12,0.35)	0.731
	Growth Areas vs Outer	-0.05(-0.78,0.68)	1.000		2016 vs 2014	-0.03(-0.26,0.21)	1.000
2012	Middle vs Inner	-0.28(-0.93,0.37)	0.831	Middle	2012 vs 2008	0.25(0.08,0.42)	0.000
	Outer vs Inner	-0.14(-0.86,0.58)	0.996		2014 vs 2008	0.63(0.46,0.79)	0.000
	Growth Areas vs Inner	-0.39(-1.14,0.36)	0.670		2016 vs 2008	0.74(0.57,0.91)	0.000
	Outer vs Middle	0.14(-0.48,0.76)	0.992		2014 vs 2012	0.38(0.21,0.54)	0.000
	Growth Areas vs Middle	-0.11(-0.76,0.54)	0.998		2016 vs 2012	0.49(0.32,0.65)	0.000
	Growth Areas vs Outer	-0.25(-0.97,0.47)	0.933		2016 vs 2014	0.11(-0.05,0.28)	0.376
2014	Middle vs Inner	-0.05(-0.7,0.6)	1.000	Outer	2012 vs 2008	0.38(0.16,0.59)	0.000
	Outer vs Inner	-0.01(-0.73,0.71)	1.000		2014 vs 2008	0.65(0.44,0.87)	0.000
	Growth Areas vs Inner	-0.26(-1.01,0.49)	0.934		2016 vs 2008	0.71(0.49,0.93)	0.000
	Outer vs Middle	0.04(-0.58,0.66)	1.000		2014 vs 2012	0.28(0.06,0.49)	0.005
	Growth Areas vs Middle	-0.21(-0.86,0.44)	0.951		2016 vs 2012	0.33(0.11,0.55)	0.000
	Growth Areas vs Outer	-0.25(-0.97,0.48)	0.936		2016 vs 2014	0.05(-0.16,0.27)	0.985
2016	Middle vs Inner	0.09(-0.56,0.74)	0.999	Growth	2012 vs 2008	0.18(-0.07,0.43)	0.314
	Outer vs Inner	0.07(-0.65,0.8)	1.000		2014 vs 2008	0.46(0.21,0.71)	0.000
	Growth Areas vs Inner	-0.24(-0.99,0.51)	0.955		2016 vs 2008	0.45(0.2,0.7)	0.000
	Outer vs Middle	-0.02(-0.64,0.6)	1.000		2014 vs 2012	0.28(0.04,0.51)	0.011
	Growth Areas vs Middle	-0.33(-0.98,0.32)	0.697		2016 vs 2012	0.27(0.04,0.51)	0.015
	Growth Areas vs Outer	-0.31(-1.03,0.41)	0.833		2016 vs 2014	-0.01(-0.24,0.23)	1.000
